# Insights Into the Emerging Entity of HER2-Low Breast Cancer

**DOI:** 10.1155/2024/2853007

**Published:** 2024-06-13

**Authors:** Georges El Haddad, Ernest Diab, Michel Hajjar, Maroun Aoun, Farid Mallat, Ziad Zalaquett, Hampig-Raphael Kourie

**Affiliations:** Hematology-Oncology Department Hôtel-Dieu de France University Hospital Saint Joseph University of Beirut, Beirut, Lebanon

**Keywords:** breast cancer, FISH, HER2, HER2-low, immunohistochemistry, molecular profiling

## Abstract

Human epidermal growth factor receptor 2 (HER2)-low breast cancer (BC) is a subtype of BC that has been recently recognized as a separate clinical entity with distinct clinical and molecular characteristics. It is defined by a low level of HER2 protein expression, which distinguishes it from other more aggressive BC subtypes. Early studies suggest that it may have a more favorable prognosis than HER2-positive BC, as it is less likely to spread to other parts of the body and may be more responsive to standard BC treatments such as chemotherapy, radiation therapy, and hormone therapy. Given the relative new emergence of HER2-low BC, there is still much to be learned about this subtype; ongoing research is focused on identifying the underlying genetic mutations that contribute to HER2-low BC as well as developing targeted therapies that can improve outcomes for patients with this disease. This review is aimed at summarizing the current clinical knowledge on HER2-low BC, with the aim of creating a better understanding of this entity and paving the way for potential interventions and a new standard of care.

## 1. Introduction

Breast cancer (BC) is the most prevalent cancer worldwide, with 2.3 million women diagnosed in 2020 and 685,000 deaths from the disease according to the World Health Organization (WHO) [1]. Despite being historically considered a disease of developed countries, over half of BC diagnoses and two-thirds of fatalities were recorded in the less developed regions of the world in 2020 [2]. With its increasing prevalence among women and men and its huge impact on global health, BC has become a crucial topic for medical practitioners. It is a perpetually modified structure whose clinical understanding is continuously evolving; medical professionals must therefore keep up with the most updated advancements in BC diagnostic methods, treatments, and management, with the goal of providing optimal care and improving patient outcomes.

An immunohistochemical classification is currently used worldwide to stratify BC patients, with each group having different treatment, prognosis, and management guidelines. The classification divides patients into four distinct categories, depending on hormonal (estrogen and progesterone) receptor expression, human epidermal growth factor receptor 2 (HER2) expression, and the Ki67 marker [3]. HER2 is a membrane tyrosine kinase amplified in numerous human cancers, especially BC. Its overexpression has been linked to increased tumor cell proliferation, invasion, and antiapoptosis [4]. While it was initially an indicator of poor prognosis, the role of HER2 has dramatically evolved over the past three decades into an indication for targeted therapy when overexpressed in tumor cells [5–7]. HER2-low BC is a new clinical entity that has recently emerged, rendering new targeted therapeutic options available [8].

## 2. Definition

HER2 status is determined using conventional molecular techniques based on the estimation of HER2 gene (ERBB2) amplification by fluorescent in situ hybridization (FISH) and the assessment of HER2 protein overexpression by immunohistochemistry (IHC) [9]. An expert group from the College of American Pathologists (CAP) and the American Society of Clinical Oncology (ASCO) recommended an algorithm for evaluating the outcomes of these molecular approaches [10–12]. The most recent version in 2018 streamlined the IHC and FISH criteria into a binary classification system as either HER2-positive or HER2-negative [13]. A tumor is considered HER2-positive if it exhibits full and intense circumferential HER2 IHC staining in more than 10% of cells (score 3+) and/or if the gene is amplified with an average HER2 gene copy number of more than 4.0 signals per cell and a HER2/CEP17 ratio of more than 2 [12]. Consequently, the definition of HER2 equivocal status was taken down, and the number of HER2 FISH-negative cases significantly rose while the number of HER2 FISH-positive patients slightly decreased [14].

Considering this classification, 80%–90% of BC tumors are HER2-negative and 10%–20% are positive [15]. However, there is a lot of variation in how hormone receptors (HRs) and HER2 are expressed in HER2-negative tumors. For instance, HER2-negative tumors that exhibit some HER2 protein expression by IHC (e.g., 1+ or 2+ and lack of ERBB2 amplification by ISH methods) are recently being classified as HER2-low [16]. Recent reports demonstrated that a significant number (between 45% and 55%) of tumors that were previously categorized as HER2-negative cancers are actually HER2-low and should be considered as a separate entity because they exhibit different characteristics than HER2-negative tumors [17, 18].

HER2-low BC tumors are not classified into the HER2-enriched subtype, but they may be categorized into other intrinsic subtypes such as Luminal A or basal-like, based on their gene expression patterns. Since more than 50% of BCs are defined as HER2-low BCs, it is important to cover the mapping of HER2-low BCs to the intrinsic subtypes. Moreover, it was found that the proportion of HER2-low was higher in HR-positive disease (65.4%) than TNBC (36.6%). It was also found that within HR-positive disease, ERBB2 and luminal-related genes were more expressed in HER2-low than HER2-negative [19]. Another study identified 410 HER2-low tumors (336 with positive hormonal receptor status and 74 with negative HR status). They found that HER2-enriched tumors were more recurrent in HER2-low/HR− and HER2-low/HR+ subtypes, compared to HER2-negative/HR− and HER2-negative/HR+ subtypes, respectively (13.7% versus 1.6% and 1.2% versus 0.5%, respectively) [8]. There is a strong rationale that, also in HER2-low tumors, different intrinsic subtypes may explain different clinical behavior, as this has been already seen in other BC subtypes.

## 3. Epidemiology

According to GLOBOCAN, the incidence, prevalence, and mortality of BC worldwide were, respectively, 11.7%, 15.4%, and 6.9% (both sexes, all ages, 2020) [2]. Stratified by its molecular type, Luminal A BC (ER+, HER2−, and Ki67 < 14%) represents around 50%–60% of cancers [20], while Luminal B (ER+, HER2+, and Ki67 > 14%), triple negative BC (TNBC) (ER−, PR−, and HER2−), and HER2+ BC (HER2+, ER−, and PR−) represent, respectively, 15%–20% [21], 12%–17% [22], and 15%–20% [23] of BC diagnoses. With the new HER2-low entity, 65% of luminal BCs and 33% of TNBC will be considered HER2-low, summing up to 50%–60% of BCs [8, 17, 19, 24].

## 4. Clinical and Histological Characteristics

HER2-low breast tumors have distinct clinical and histological features when compared with HER2-zero and HER2-positive BC. HER2-low tumors have a higher HR-positive status than HER2-zero (87.4% vs. 66.7%, *p* < 0.001) and HER2-positive (87.4% vs. 58.4%, *p* < 0.001) tumors [9]. Several other studies have also revealed the higher prevalence of positive HR in HER2-low tumors when compared with HER2-zero and HER2-positive tumors [25–27]. Zhang et al. further reported a significantly lower Ki67 expression level in HER2-low tumors when compared with HER2-zero [9], with other studies reporting similar conclusions [27, 28]. Moreover, HER2-low tumors are more likely to exhibit histological Grade 2 than HER2-zero (59.3% vs. 34.4%, *p* < 0.001) and HER2-positive (59.3% vs. 39.6%, *p* < 0.001) tumors. Conversely, HER2-low BC was less likely to express histological Grade 3 than both HER2-zero (35.5% vs. 55.6%, *p* < 0.001) and HER2-positive (35.5% vs. 57.4%, *p* < 0.001) [9].

Clinically, HER2-low tumors appear to be more common in older and male patients and involve more axillary lymph nodes than HER2-negative cancers [19]. Liu et al. conducted a retrospective study on over 4000 patients with HER2-zero and HER2-low tumors and compared their clinical characteristics: no significant difference was found in the tumor stage at diagnosis when classifying tumors from Stage I to Stage III (*p* = 0.188). However, after subdividing the tumor by the TNM classification, it was revealed that HER2-low tumors were more frequently diagnosed at T1, while HER2-zero tumors were more frequently diagnosed at T2 (*p* = 0.025). HER2-low tumors were also diagnosed at a slightly older age than HER2-zero patients (median age, 53 years vs. 51 years, *p* < 0.001) [29].

## 5. Evolution

Having defined HER2-low BC using the IHC score, thus creating a new subdivision in the TNBC and HR-positive BC, studies were conducted to determine the evolution of the HER2 expression in both those groups. Most of the analyses have underlined a discordance in the HER2 expression level between the primary tumor and the corresponding biopsy [30]. It has been found that, regardless of the molecular subtype (Luminal A/B or TNBC), HER2 status shows inconsistency due to the switch in its expression [31]. Most of the cases transition between HER2-low and HER2-zero, with the IHC score of the HER2-low decreasing during a relapse biopsy compared to the primary tumor and transitioning it to a HER2-zero status, while the IHC score of the HER2-zero primary BC tends to increase and switch to a HER2-low status. The numbers show different percentages of switch depending on the molecular subtype. The transition rates rise to 21.42%, with most of them switching from HER2-low to HER2-zero, while other studies affirm that the discordance rates are higher in HR-positive tumors rather than TNBC (45.5% vs. 36.7%) [32, 33]. The instability in HER2 expression may offer new therapeutic opportunities; this conversion therefore highlights the importance of continuous HER2 expression testing in relapse biopsies while adapting the treatment accordingly.

## 6. Genetics

Numerous studies have investigated the genomic profile of HER2-low breast tumors. Berrino et al. reported that the most common mutations found were *PIK3CA* (31%), *GATA3* (18%), *TP53* (17%), and *ERBB2* (8%). They further found multiple somatic mutations in genes associated with hereditary BC including *BRCA2*, *BARD1*, and *PALB2* [34]. Zhang et al. noted a stark contrast between the genomic profiles of HER2-low, HER2-zero, and HER2-positive tumors. HER2-low tumors had more mutations in *GATA3* (*p* < 0.001), *CBFB* (*p* < 0.001), *PTEN* (*p* < 0.001), and *AKT1* (*p* < 0.001) compared with HER2-positive tumors. They also had more mutations in *MAP3K1* (*p* < 0.05), *PIK3CA* (*p* < 0.05), *CBFB* (*p* < 0.05), and *ARID1A* (*p* < 0.05) than HER2-zero. HER2-low genomic profiles also displayed higher mutation rates in the PI3K-Akt pathway than either HER2-zero (*p* < 0.001) or HER2-positive (*p* < 0.01). However, they showed significantly less copy number amplification in genes such as the retinoic acid receptor alpha (*RARA*, *p* < 0.001) and *CKD12* (*p* < 0.001) and a much lower mutation rate in *TP53* when compared with HER2-positive tumors. The authors also noted lower mutation rates in *TP53* (*p* < 0.01) *TERT* (*p* < 0.05), *GALNT12* (*p* < 0.05), *CARD11* (*p* < 0.05), *TRRAP* (*p* < 0.05), checkpoint factors (*p* < 0.01), and p53 pathway (*p* < 0.05) in HER2-low relative to HER2-zero [9].

In addition, Schettini et al. conducted a PAM50 analysis on over 1300 tumors classified as either HER2-low or HER2-zero. In the 55 genes analyzed, they found that about 60% of genes were differentially expressed between the two groups. HER2-low tumors showed an upregulation of luminal-related genes including *BCL2*, *BAG1*, *FOXA1*, and *ESR1*, while genes related with proliferation (*CCNE1*, *CCNB1*, *MYBL2*, *MKI67*, and *MELK*) and tyrosine kinase receptors (*EGFR*, *FGFR4*) were downregulated [19].

Berrino et al. further classified HER2-low breast tumors into four subgroups based on genomic and histologic profiles. Group A had high mutational burden, Ki67 expression, and PD-L1 receptor expression, indicating a high immune infiltration phenotype. Group B comprised exclusively luminal tumors. Group C, mainly Luminal A tumors, exhibited low Ki67 expression and upregulated genes related to stromal adhesion. Group D had high progesterone receptor expression and intermediate Ki67 levels, with a high prevalence of PIK3CA mutations (50%). Relapse-free survival rates were compared: Group B showed a significantly higher rate (24%), while Groups A, C, and D had rates of 10%, 8%, and 15%, respectively. However, statistical significance was limited due to small sample sizes and treatment heterogeneity; further studies are needed to confirm clinical significance [34].

## 7. Treatment

### 7.1. Trastuzumab Deruxtecan (T-DXd)

Several studies have investigated the efficacy of T-DXd in the treatment of HER2-low BC. In the pivotal DESTINY-Breast04 trial, Modi et al. conducted a study on 557 patients with metastatic BC; 373 were assigned to the T-DXd group where they received T-DXd once every 3 weeks at a dose of 5.4 mg/kg, while 184 were assigned to the physician's choice where they received chemotherapy in accordance with the NCCN guidelines. HER2-low patients treated with T-DXd had a median progression-free survival (PFS) of 9.9 months, compared to 5.1 months in patients who underwent chemotherapy (*p* < 0.001). Overall survival (OS) was also significantly higher in people treated with T-DXd (23.4 months vs. 16.8 months, *p* < 0.001) [35]. Further subdivision found that patients with HR-positive HER2-low had a higher median PFS (10.1 months vs. 5.4 months, *p* < 0.001) and OS (23.9 months vs. 17.5 months, *p* = 0.003). HR-negative HER2-low patients also benefited from T-DXd, with a higher median PFS (8.5 months vs. 2.9 months) and a higher OS (18.2 months vs. 8.3 months) [35]. Numerous other trials are actively evaluating the potential of T-DXd in the treatment of HER2-low BC, as the treatment could become a new standard of care for these patients.

Modi et al. also reported a decreased incidence of Grade 3 adverse effects in T-DXd patients compared with chemotherapy (52.6% vs. 67.4%), with the most common of them being neutropenia (13.7%), anemia (8.1%), and fatigue (7.5%) [35]. Shimomura et al. found that T-DXd was not associated with a clinically relevant prolongation of QTc [36].

### 7.2. Neoadjuvant Chemotherapy

Neoadjuvant chemotherapy is often administered to patients with metastatic HER2-low BC. Several studies have compared the pathological complete response (PCR) between patients with HER2-low and HER2-zero BC. Shao et al. found that the PCR rate was significantly lower in HER2-low patients (24.3% vs. 36.4%, *p* = 0.035). PCR was also lower in HR+ HER2-low patients when compared with HR+ HER2-zero (18.7% vs. 32.1%, *p* = 0.035) [25]. Other studies have reported similar findings regarding the PCR rates of HER2-low vs. HER2-zero [26, 27, 37, 38]. HER2-low patients had, however, a higher OS than HER2-zero patients, with one study reporting a 5-year OS of 92.4% vs. 84.1% for HER2-zero (*p* < 0.001). The 5-year disease-free survival (DFS) was higher as well, with 77.8% for HER2-low vs. 71.6% for HER2-zero patients (*p* = 0.002) [26, 39, 40].

### 7.3. CDK4/6 Inhibitors

Shao et al. compared the clinical efficacy of CDK4/6 inhibitors between HER2-low and HER2-zero patients. Both overall response rate and disease control rate (DCR) were compared, but no statistically significant difference was found (28.6% vs. 41.7%, *p* = 0.36, and 76.2% vs. 79.2%, *p* = 0.811, respectively). PFS was also similar between the two groups at 14.1 months for HER2-low and 16.2 months for HER2-zero (*p* = 0.263). However, it was found that patients who received CDK4/6 inhibitors as a third-line therapy or above had a significantly lower PFS (10.8 months) than those who were administered CDK4/6 inhibitors as a first- (22.6 months) or second-line (16.5 months) treatment (*p* = 0.019) [41]. Douganiotis et al. conducted a similar study on 191 patients with different levels of HER2 expression and found that they were equally effective between the 3 subgroups (HER2-zero, HER2 1+, and HER2 2+ with ISH−). They also found that the PFS was higher in patients who took aromatase inhibitors instead of selective estrogen receptor degraders (SERDs) [42]. Carlino et al. also conducted a similar study on 165 patients treated with palbociclib and endocrine therapy in 6 Italian hospitals, but no statistically significant difference in OS and DFS was found [43].

## 8. Future Therapies

### 8.1. Immunotherapy

Li et al. compared the clinicopathological features and biomarker expression of patients with HER2-low and HER2-zero BC. It was found that PD-L1 expression was similar among both groups, with 32% of cancers expressing PD-L1 in immune cells [44]. Another study by Hamilton et al. compared the DCR between HER2-positive and HER2-low patients treated with T-DXd and nivolumab. In both groups, the DCR (90% for HER2-positive vs. 75% for HER2-low) was similar to that of patients treated with T-DXd alone in previous studies. They also had an acceptable level of adverse effects, with 44% of patients experiencing adverse events of Grade 3 or higher [45]. Determining whether the addition of immunotherapy to T-DXd provides benefits to patients necessitates further investigation through extended follow-up and additional studies.

### 8.2. Gold Nanoparticles

Bloise et al. conducted an experimental study in which they linked gold nanoparticles with trastuzumab in an attempt to increase the selectivity and specificity of trastuzumab towards cells expressing the HER2 receptor, hence increasing the treatment efficacy and decreasing the side effects. They found that in vitro MCF-7 cells (cells expressing HER2-low) treated with the nanoparticle-linked trastuzumab had a lower viability than cells treated with free trastuzumab (20% after 3 days of culture) [46]. This study could open the door for exciting new treatments which involve nanoparticles to increase the selectivity of trastuzumab.

### 8.3. *α*-Amanitin-Conjugated Trastuzumab (T-Ama)

The heterozygous loss of the fragment p of chromosome 17 (containing the tumor suppressor p53) is one of the most common mutations in BC; its loss therefore increases the tumor's progression [47]. However, this region also includes *POLR2A*, a gene responsible for encoding a subunit of an RNA polymerase. The absence of this subunit and therefore a homozygous deletion of this gene would therefore result in the tumor cell's death and slow tumor progression [48]. Li et al. have conducted an experimental study in which they have linked trastuzumab with a *α*-amanitin, a specific inhibitor of *POLR2A*. This combination (called T-Ama) has shown a higher efficacy in treating patients who had a 17p-mutated HER2-low BC [48].

A summary of the mentioned studies tackling HER2-low BC treatment as well as their outcomes is shown in Table 1.

### 8.4. Financial Considerations

Cancer treatment can be extremely expensive, especially for patients in third-world countries. Hence, it is important to assess the cost–utility ratio (CUR) of such a treatment. As stated previously, T-DXd has proven to be effective in treating HER2-low BC in multiple studies. Lang, Wu, and Liu have evaluated and compared the incremental CUR (ICUR), a tool used to determine the cost of a treatment per quality-adjusted life years, between T-DXd and neoadjuvant chemotherapy in patients with HER2-low BC: they found that the ICUR of T-DXd was $346,571.8/QALY whereas chemotherapy, which is not particularly effective in HER2-low BC, had an ICUR of $337,789.4/QALY. It is important to note that in the United States, the threshold for a cost-effective ICUR is $150,000/QALY which is significantly lower than the ICUR obtained with T-DXd. T-DXd cannot therefore be considered a cost-effective treatment for HER2-low BC patients at its current price [49]. Other studies have found similar results as to the cost-effectiveness of T-DXd in the United States and China [50, 51]. It is important to note, however, that the treatment price and reimbursement models widely differ between countries and what is considered cost-effective in a country may be different in another country.

### 8.5. Prognosis

After subdividing the HER2-negative into HER2-low and HER2-zero, many studies were conducted to determine if this subdivision leads to a better understanding of the prognosis of BC. Not only do they differ in terms of histological grading, Ki67 expression, they also have a slightly different prognosis depending on the HR status [52]. The studies compare DFS and OS between HER2-zero and HER2-low groups in the population with HR-positive and HR-negative. In early-stage luminal disease, there is a significant favorable outcome in the prognosis of HER2-low compared to HER2-zero in patients with a high genomic risk [53].

Most of the studies have shown that patients with HER2-low BC tend to have a better and superior DFS and OS compared to HER2-0 in the HR-positive group, whereas they have not concluded any significant improvement in the prognosis between HER2-low and HER2-zero in the HR-negative population [54]. A better DFS after 5 years is predicted with a 69% reduced risk compared to HER2-zero, but the results were not conclusive regarding the OS [54]. The distinctive gene expression profiles between HER2-zero and HER2-low tumors within HR-positive disease further support the notion that HR-positive/HER2-low tumors represent a distinct biological entity. The underlying reasons for the better long-term DFS in HR-positive/HER2-low BC remain unclear, but potential factors may include the presence of HER2-enriched subtypes, reduced aggressiveness, and complex interactions between HR and HER2 [54]. In contrast, the results are actually the opposite in tumors with a high Ki67 index since the survival rate is poorer for HER2-low BC [55].

Conflicting findings have been the reason that the prognosis of HER2-low BC remains a subject of debate, but the majority agrees that even though the clinicopathological characteristics between HER2-low and HER2-zero were similar between HR-positive and HR-negative BC, the better prognosis of HER2-low tumors has only been consistent in HR-positive patients.

### 8.6. Future Directions

We currently face two main challenges with HER2-low BCs; first is understanding its biology and second is the formulation a stable definition of the disease. Although significant efforts have been made to understand the biology of low HER2 BC, its biological complexity and heterogeneity remain poorly understood. Furthermore, there is still no firm evidence that HER2 levels in BC are an independent prognostic factor and a distinct biological/clinical entity. Further studies focusing on clinical and molecular biomarkers that could potentially influence the response of HER2-low BC to new antibody–drug conjugates (ADCs) should be considered.

HER2-low BC's definition as of today is largely based on the inclusion criteria of patients enrolled in the previously mentioned clinical trials. As these inclusion criteria vary between the different studies, there is no adequate representation of the ideal target population. Preliminary results from a Phase II study (DAISY) reported a response rate of 30.6% to T-DXd treatment in BC patients with HER2 IHC score of 0 (vs. 69.1% in HER2-positve tumors, 33.3% in HER2-low tumors) [56]. The DESTINY-Breast06 trial (NCT04494425), which is designed to evaluate the efficacy, safety, and tolerability of T-DXd in metastatic HER2-low/HR-positive BC patients whose disease has progressed on endocrine therapy in the metastatic setting, is actively recruiting BC patients with HER2-low BCs and HER2 IHC expression of > 0 and < 1+. This study has important implications for determining the lower threshold level of HER2 expression needed to benefit from an ADC therapeutic approach, as well as the clinical significance of current tests for distinguishing low HER2 from HER2-null BC. Future studies correlating patient response to new ADCs and HER2 expression in clinical trial data sets would also be very useful to better define HER2-low BC. We believe that the definition of HER2-low BC will continue to evolve based on the development of new drugs, the results of future clinical trials, the development of more sensitive and reliable testing methods, and our understanding of HER2-expressing BCs.

## 9. Conclusion

In conclusion, HER2-low BC represents a distinct clinical entity with unique molecular, genetic, and histological features. While initial studies suggest a more favorable prognosis compared to HER2-positive BC, ongoing research is essential to fully understand its clinical significance and develop targeted therapies. The identification of HER2-low subtype is gradually increasing in importance, as it creates a foundation for a new BC classification and better therapeutic strategies. Numerous studies have shown insights into its genetic profile, treatment response, and prognostic implications, underlying the need for personalized interventions and continued investigation. These findings, along with future others, are aimed at better characterizing this novel clinical subgroup and improving patient outcomes.

## Figures and Tables

**Table 1:** The mentioned studies on HER2-low BC treatment and their respective outcomes.

**Manuscript reference**	**Authors**	**Year**	**Study type**	**Treatment**	**Primary endpoint**	**Secondary endpoint**
[35]	Modi et al.	2022	Phase 3 clinical trial	Trastuzumab deruxtecan	PFS: T-DXd>chemotherapy (9.9 months vs. 5.1 months; *p* < 0.001) OS: T-DXd>chemotherapy (23.4 months vs. 16.8 months; *p* = 0.001)	Grade 3 adverse events: T-DXd<chemotherapy(52.6% vs. 67.4%)
[36]	Shimomura et al.	2023	Clinical trial	Trastuzumab deruxtecan	T-DXd is not associated with a clinically relevant prolongation of QT	T‐DXd ORR = 43.1% T‐DXd DCR = 83.4%
[25]	Shao et al.	2022	Retrospective study	Neoadjuvant chemotherapy	In all patients, PCR HER2-low<HER2-zero(24.3% vs. 36.4%; *p* = 0.032) In HR+ patients, PCR HER2-low<HER2-zero(18.7% vs. 32.1%; *p* = 0.035) In HR− patients, no statistically significant PCR difference was found	3-year DFS and OS between HER2-low and HER2-zero: No statistically significant difference
[26]	Kang et al.	2022	Retrospective study	Neoadjuvant chemotherapy	In all patients, PCR HER2-low<HER2-zero(9.8% vs. 14.7%; *p* = 0.003) In HR+ patients: No statistically significant PCR difference was found (*p* = 0.4) In HR− patients, no statistically significant PCR difference was found (*p* = 0.3)	5-year OS HER2-low>HER2-zero (92.4% vs. 84.1%; *p* < 0.001) 5-year DFS HER2-low>HER2-zero (77.8% vs. 71.6%; *p* = 0.002)
[38]	Cherifi et al.	2022	Retrospective study	Neoadjuvant chemotherapy	No statistically significant PCR difference between HER2 1+ and HER2 2+ (*p* = 0.77)	OS HER2 2+>HER2 1+ (HR = 0.31; *p* = 0.042) DFS HER2 2+>HER2 1+ (HR = 0.41; *p* = 0.037)
[39]	Wang et al.	2022	Retrospective study	Neoadjuvant chemotherapy	PCR HER2-low<HER2-zero (12.9% vs. 16.36%)	18-month DFS HER2-low>HER2-zero (HR = 0.16; *p* = 0.04)
[27]	Alves et al.	2020	Retrospective study	Neoadjuvant chemotherapy	PCR HER2-low<HER2-zero (14.6% vs. 29.0%; *p* = 0.15)	DFS: No statistically significant difference OS: No statistically significant difference
[37]	de Nonneville et al.	2022	Retrospective study	Neoadjuvant chemotherapy	PCR HER2-low<HER2-zero (23% vs. 30%; *p* = 0.0013) In HR+ patients, PCR HER2-low<HER2-zero(10% vs. 16%; *p* = 0.046) In HR− patients, no statistically significant PCR difference was found (46% vs. 42%; *p* = 0.346)	3-year DFS: No statistically significant difference (*p* = 0.742)
[40]	Zhou et al.	2023	Retrospective study	Neoadjuvant chemotherapy	PCR HER2-low vs. HER2-zero (16.7% vs. 18.9%; *p* = 0.374)	OS HER2-low>HER2-zero (HR = 0.302; *p* = 0.001) DFS HER2-low>HER2-zero (HR = 0.573; *p* = 0.066)
[28]	Shi et al.	2023	Retrospective study	Neoadjuvant chemotherapy	PCR: No statistically significant difference	Pathological tumor size HER2-low>HER2-zero(21.6 vs. 17.8 mm; *p* = 0.028)
[41]	Shao et al.	2022	Retrospective study	CDK4/6 inhibitors	ORR HER2-low vs. HER2-zero (28.6% vs. 41.7%; *p* = 0.360) DCR HER2-low vs. HER2-zero (76.2% vs. 79.2%; *p* = 0.811) PFS HER2-low vs. HER2-zero (14.1 months vs. 16.2 months; *p* = 0.263)	PFS palbociclib 1st-line therapy>2nd-line therapy>3rd-line therapy (22.6 months, 16.5 months, 10.8 months; *p* = 0.019)
[42]	Douganiotis et al.	2022	Retrospective study	CDK4/6 inhibitors	PFS HER2-zero>HER2 1+>HER2 2+ (3.35.years vs. 2.18 years vs. 1.74 years; *p* = 0.477)	PFS aromatase inhibitor>fulvestrant (2.99 years vs. 1.33 years; *p* < 0.001)
[43]	Carlino et al.	2022	Retrospective study	CDK4/6 inhibitors	PFS HER2-low<HER2-zero (19 months vs. 23 months; *p* = 0.2) 48-month survival probability HER2-low<HER2-zero (62% vs. 51%; *p* = 0.1)	
